# Trends of inequities in healthcare seeking behavior for diarrhea, fever, and ARI symptoms among women in reproductive age groups for their under-five children in Ethiopian: A multilevel Analysis of EDHS Surveys from 2000 to 2016

**DOI:** 10.1371/journal.pone.0318651

**Published:** 2025-04-01

**Authors:** Hailu Fekadu, Wubegzier Mekonnen, Aynalem Adugna, Helmut Kloos, Damen Hailemariam

**Affiliations:** 1 Department of Public Health, Arsi University College of Health Science, Assela, Ethiopia; 2 School of Public Health, Addis Ababa University College of Health Science, Addis Ababa, Ethiopia; 3 Department of Geography, Planning and Environmental Sonoma state University, Sonoma, San Francisco, California, United States of America; 4 Department of Epidemiology and Biostatistics, University of California, San Francisco, California, United States of America; Bahir Dar University College of Medical and Health Sciences, ETHIOPIA

## Abstract

**Background:**

Ethiopia is one of the developing countries with the highest inequity in the healthcare seeking behavior for under-five children. Despite this fact, not much is known about the trend of inequity, in healthcare seeking-behavior for symptoms of diarrhea, fever and acute respiratory infections (ARIs) among under-five children.

**Objective:**

This study aimed to measure trends of inequity in healthcare seeking -behavior for diarrhea, fever, and ARI symptoms and its determinant factors among under-five children in Ethiopia.

**Methods:**

Data from 2000, 2005, 2011, and 2016 Ethiopian Demographic and Health Surveys (EDHSs) were analyzed using the 2019 updated version of the WHO’s Health Equity Assessment Toolkit (HEAT) software. Five equity dimensions were used to disaggregate datasets on healthcare seeking behavior for diarrhea, fever, and ARI symptoms: Based on wealth status, education, place of residence, sex of the child and administrative regions. Second, summary measures such as: equity gaps, equity ratios, population attributable fraction (PAF), population attributable Risk (PAR), absolute concentration index (ACI) and relative concentration index (RCI), was used. The concentration curve and horizontal inequity indices were used to evaluate the wealth-related disparities. To measure the determinants a multilevel logistic regression with 95% confidence interval was employed.

**Result:**

This study showed remarkable improvement in healthcare seeking behavior for symptoms of diarrhea, fever and ARI among under-five children between 2000 and 2016. The increases are more, from the poorest subgroups compared to the wealthiest. The absolute percentage point changes (or healthcare seeking rate changes) between 2000 and 2016 among the poorest quintile of households are at least twice that of the wealthiest quintile for symptoms of diarrhea. However, significant disparities between the rich and the poor persist for the majority of the indicators examined for the three childhood morbidities. Moreover, horizontal inequity indices and the concentration curve both point out to the existence of pro-rich inequity in healthcare seeking behavior for under five children. In the multilevel analysis various demographic, parental and household characteristic show an association with healthcare seeking behavior for symptoms of diarrhea, fever and ARIs.

**Conclusion:**

A promising trend is observed in healthcare seeking behavior for diarrhea, fever and ARI symptoms among under-five children. Faster progress in use of healthcare services among the poor than the wealthy in Ethiopia would potentially result in elimination of inequities and rapid improvement in health among the poor. Intervention programs that focused on the underprivileged, while also taking into account the wealthier sub-groups.

## Background

Globally, 4.9 million children died before turning five years in 2023. Nearly half of which were newborns. Almost most of these deaths were concentrated in sub-Saharan Africa and Southern Asia [1]. Ethiopia is one of the Sub Saharan Africa (SSA) countries with the highest rate of under-five mortality [2]. The recent Ethiopian Demographic and Health Survey (0) report states that the country’s under-five mortality rate was 67 deaths for every 1,000 live births [3]. Sub Saharan Africa (SSA) bears the highest burden of under-five mortality globally, with nearly 56% of global child deaths concentrated in this region. Common causes of child mortality, such as malnutrition, pneumonia, diarrheal diseases, and malaria, are exacerbated by poor healthcare infrastructure, inequitable access to treatment, and systemic barriers to healthcare-seeking behavior. Ethiopia, while having made significant progress in reducing under-five mortality from 123 deaths per 1,000 live births in 2005–67 in 2016, still faces significant challenges [4]. Ethiopia’s health-seeking behaviors are comparable to other low-resource SSA countries like Nigeria and Niger, which also report high child mortality rates but lag behind in reaching universal healthcare coverage [1,2]. But, Ethiopia lags behind countries like Ghana and Rwanda, where government-led insurance schemes and decentralized healthcare have positively impacted child health outcomes [1].

In Ethiopia, Preterm birth complications, malaria, tetanus, meningitis, pneumonia, birth asphyxia, and measles are the leading causes of death for under- five children []. The majority of these deaths could have been prevented by using equitable and widely available treatment methods, such as oral rehydration for diarrheal illnesses, antibiotics for acute respiratory infections, and the right medication for malaria [5,6]. However, a large number of children continue to die without obtaining sufficient care and treatment, never reaching a health facility, or because of delays in seeking care [6]. Delayed or lack of healthcare for common but potentially severe conditions like diarrhea, fever, and ARI can lead to higher mortality rates among under-five children. Prompt treatment is critical to prevent complications and fatalities. According to WHO estimates, caregivers who seek appropriate and timely care could reduce child mortality by 20%. And yet, only a small percentage of children in Ethiopia receive proper treatment due to poor healthcare seeking behavior [7]. Overall, in the five years before the 2016 survey, only thirty percent of children under five with acute respiratory infection symptoms, 35% with fever, and 43% with diarrhea were brought to a health facility [3]. However, equity in the health sector has long been regarded as one of the main objectives of the healthcare system [8,9]. Although the poor require more healthcare services than the wealthy, they also tend to seek and use services less frequently, and as a result, they have higher rates of morbidity and mortality [9,10]. The reasons are poor individuals often live in environments with inadequate sanitation, unsafe drinking water, and poor housing conditions, increasing their exposure to infectious diseases like diarrheal illnesses, malaria, and respiratory infections. Moreover, the poor has limited preventive care such as vaccinations, regular check-ups, and early screenings, reducing their disease burden. There is a growing belief that inequities in child health outcomes are unfair and injustices that can be avoided. In particular, rather than reflecting personal preference or behavior, disparities in child health are thought to primarily reflect underlying imbalances in the distribution of wealth, resources, and social privilege in society. Therefore, efforts must be made to address disparities in the burden of morbidity and mortality among children from low-income families [11]. In recent decades, Ethiopian’s health status has significantly improved [12,13]. For instance, Ethiopia significantly reduced the under-five mortality rate from 205 deaths per 1,000 live births in 1990 to 55 per 1,000 in 2019, achieving Millennium Development Goal 4 three years ahead of schedule [14,15]. Despite these advancements, significant inequities exist in child health outcomes and in healthcare seeking behavior among population subgroups [16]. Due to the burden of highly prevalent diseases like diarrhea, fever, pneumonia, and malaria, Ethiopia continues to have high rates of childhood morbidity and mortality. For instance, diarrhea is a factor in over one in ten (13%) child deaths [17]. The Ethiopian government is dedicated to enhancing equity in healthcare seeking behavior through various initiatives, including the health extension program [18]. In addition, meeting the needs of Ethiopia’s rural population, which makes up 84% of the country’s total population, is a top priority in the country’s national health policy. Furthermore, the national Health Sector Transformational Plan’s primary goal is to guarantee that everyone has access to healthcare [16,19,20]. However, only 16% and 22% of households from the poorest quintile and 62% and 53% from the richest quintile, respectively, sought medical care for a child with presenting symptoms of pneumonia or diarrhea [20,21]. Furthermore, the difference between urban and rural areas is even more noticeable when it comes to mortality rates for children under five, which are up to 37% higher in rural areas. Additionally, significant regional differences in child mortality rates observed, with a more than two fold difference, between Addis Ababa and Benishangul-Gumuz [3,21,22].

Despite this, little is known about the trends of inequity in healthcare seeking behavior, and its determining factors for diarrhea, fever and symptoms of ARI in Ethiopia, particularly in rural areas and low socioeconomic subgroups. Moreover, several studies have been undertaken in various countries including in Ethiopia to investigate the care seeking pattern and relevant factors for the utilization of health care services in cases of childhood illnesses [22,23–25]. But none of them look at the trends of inequity in health seeking behavior for under -five children. Moreover, most studies conducted in Ethiopia have focused on specific local areas, relied on micro-level data, or examined only a single demographic group, such as rural populations. These studies have often overlooked important child morbidity outcomes, such as diarrhea and fever [15,26–29]. Moreover, Ethiopia’s healthcare system, though improving through initiatives such as the Health Extension Program (HEP), still faces gaps in coverage, particularly in rural areas. Comparing Ethiopia with other SSA nations highlights the urgent need for equitable healthcare policies that ensure access to preventive and curative services for under-five children, especially in remote areas [1]. The period from 2000 to 2016 is particularly important because it aligns with the Ethiopian government’s major health initiatives, such as the Health Extension Program (HEP) launched in 2003, which aimed to improve access to primary healthcare, particularly in rural areas. Investigating trends over this period helps assess the impact of these initiatives on healthcare-seeking behavior. Additionally, the period overlaps with the Millennium Development Goals (MDGs), which targeted the reduction of child mortality (Goal 4). Analyzing healthcare-seeking trends from 2000 to 2016 can show whether the efforts to meet these goals led to more equitable access to health services for common childhood illnesses. Although numerous studies have investigated health-seeking behavior, there has been limited analysis of trends in health-seeking behavior, particularly in tracking changes over time in Ethiopia. This study addresses this gap in the literature by examining how health-seeking behaviors for common childhood illnesses have evolved over time in response to policy changes and economic shifts. This study addresses a gap in the literature by exploring how health-seeking behaviors for common childhood illnesses have evolved over time, in response to both policy shifts and economic changes. By focusing on the trend from 2000 to 2016, the study provides insights into whether improvements in healthcare access have been equitable or if disparities persist.

Thus, understanding inequities in healthcare-seeking behavior is essential for promoting health equity. This involves examining disparities based on socio-economic status, geographic location, education level, and other factors. By addressing these points, the study aims to provide comprehensive insights into the factors influencing healthcare-seeking behavior for common childhood illnesses in Ethiopia, which can inform policy and programmatic interventions to improve child health outcomes and reduce inequities [30].

## Methodology

### Study setting and period

Ethiopia has a population of over 120 million, making it the second most populous nation in Africa after Nigeria [32]. And, continues to have the fastest-growing economy in the area, expanding by 6.3% in FY2020–2021. With a gross national income per capita of $960, it is also among the poorest [29]. By 2025, Ethiopia wants to be considered a lower middle-income country [32]. Ethiopia’s economy has been among the fastest growing in the world (at an average of 9.5% per year). The last eight years have seen steadily high economic growth, which has led to encouraging trends in the decline of poverty in both urban and rural areas. Human development metrics also improved as the percentage of the population living below the national poverty line dropped from 30% in 2011 to 24% in 2016. But gains in healthcare services utilization are small compared to other nations that experienced rapid growth, and inequity has gotten worse recently. Moreover, the ongoing conflicts in different regions of Ethiopia pose a threat to the recent advancements Ethiopia has made in its social and economic development [33]. Ethiopia uses a decentralized, top-down approach to provide healthcare along political hierarchies. Administratively, the nation was divided into nine regions and two city administrations during the study period, and it used a three-tiered health care delivery system. A woreda, or district healthcare system, is the lowest level and consists of a primary hospital, health centers, and their satellite health posts. While the first two sections primarily deal with curative and preventive healthcare, health posts are leaders in the delivery of preventive healthcare, particularly with regard to maternity and child care. The second tier is a general hospital, and the third tier is a specialized teaching and referral hospital that only provides curative healthcare services. Therefore, Ethiopia offers all recommended healthcare services for common childhood illnesses for under-five children using these tiers system [33]. The study was conducted from May 2021-June, 2024.

### Data sources and population

We used a cross-sectional survey data from 2000, 2005, 2011 and 2016 Ethiopian demographic health surveys (EDHSs). These EDHSs are nationally representative surveys which had been done in 9 regions and 2 administrative cities at the interval of every five years. EDHS employed stratified two-stage cluster sampling in all of the surveys. Each region was divided into rural and urban areas in order to achieve stratification. Consequently, twenty-one sampling strata in total have been established. In the initial phase, 540 Enumeration Areas (EAs) for the EDHS 2000, 540 EAs for the EDHS 2005, 650 EAs for the EDHS 2011, and 645 EAs for the EDHS 2016 were chosen at random in accordance with the size of each EA. In the second phase, each EA typically had 27–32 households chosen [3,31,34,35]. After obtaining authorization through an online request outlining the goal of our investigation, the data was obtained from the Measure DHS website (http://www.dhsprogram.com). The Kid Record (KR file) data set was used to extract the study’s variables. This study used a weighted total sample of 3098, 1432, 2134, and 1586 under five-children that were used in the EDHSs in 2000, 2005, 2011, and 2016, respectively. This led to a final sample size of 8,250 research participants. Detail explanation about sampling procedure was explained in every EDHS report [31,34–36]. The 2019 updated offline version of world health organization (WHO’s) Health Equity Assessment Toolkit (HEAT) software was used as both the source of data and tools for analysis for inequity. HEAT allows users to disaggregate health data by various social determinants (such as wealth, education, and residence) and calculate summary measures of inequality, such as rate ratios, rate differences, the concentration index, and the slope index of inequality. These metrics help in quantifying disparities within populations. The toolkit enables users to track trends over time, which is essential for this study, as it assesses changes in healthcare-seeking behavior for childhood illnesses (diarrhea, fever, and ARI) from 2000 to 2016. By analyzing data over a 16-year period, HEAT helps to identify whether inequities have been reduced or persist. A detailed explanation of the tool is provided in the Health Equity Assessment Toolkit (HEAT), software designed for exploring and comparing health inequalities across countries [36,37]. The choice of HEAT for this analysis is justified because it offers a clear, replicable approach to measure inequity in healthcare access and utilization, particularly in low-resource settings like Ethiopia.

The EDHS surveys are nationally representative and collect data on demographics, socioeconomic status, maternal healthcare and children illness. Women aged 15 to –49 were the primary data source for the surveys, while information on the children under the age of five was also collected. EDHS used the standard Demographic and Health Survey questionnaires of the DHS Program which were adapted to reflect the populations and health issues relevant to Ethiopia. After each woman provides verbal informed consent, data is gathered using both standard questionnaires and questionnaires tailored to the country’s specific context.

### Outcome variables and inequity measures

Healthcare seeking behavior for diarrhea, fever and symptoms of acute respiratory infection (ARI) were the primary outcome variables for which inequity was measured. All of these dimensions have a lot of importance for children health and economic development. Children with diarrhea and symptoms of ARI and fever were identified in the EDHS data through their mothers’ reporting whether the child had cough or fever and or diarrhea within the two weeks before the survey conducted. Two outcome variables were identified: “mother sought medical care for her child with diarrhea” and “mother sought medical care for her child with fever or symptoms of ARI.” The outcome variables were quantified and assigned a code of “1” for seeking medical care and “0” for not seeking medical care. It should be noted that the definition of medical care included treatment from public hospitals and clinics, private hospitals and clinics, and hospitals and clinics run by non-profit organizations. Conversely, care from unlicensed physicians, private pharmacies, and not receiving any care were classified as not using medical care. There were three categories for the mother’s educational status: no education, primary education, secondary education, or higher education. The richest, rich, middle, poor, and poorest people are the five categories into which the EDHS divides the wealth index, which it uses as a proxy measure of economic status.

Principal component analysis (PCA), which is explained in detail here [38] is used in EDHS to construct the wealth index, which is then deemed comparable across all survey years based on household assets and ownership. The residence was categorized as both rural and urban. The sub-national regions comprised the two city administrations and the nine regions and sub-national region segregations did not display the sequence.

Tables and figures for each of the four Ethiopia Demographic and Health Surveys (EDHS) periods were used to illustrate the trend of inequity for healthcare seeking behavior. The disaggregation included the computed point estimates with a corresponding 95% Confidence Interval (CI).

### Analysis of the data

Data analysis was conducted using the WHO’s Health Equity Assessment Toolkit (HEAT) software, which was updated in 2019 [36]. Datasets were analyzed and disaggregated using the five equity stratifiers: economic status, maternal education, place of residence, sex of the child, and subnational regions. Six summary measures of health inequity were used to present these: absolute concentration index (ACI), relative concentration index (RCI), population attributable fraction (PAF), population attributable risk (PAR), ratio (R), difference (D), and population attributable fraction (PAF) and all selected from a pool of 15 measures that the software provided [36]. These measures were chosen because they have broader use in healthcare inequity research [37]. In order to gain a deeper understanding of the disparities in health care seeking behavior for their children, as advised by the World Health Organization, equity studies computed both simple and complex summary measures for each equity stratum. In short, ACI, RCI, (PAF), and (PAR) are complex measures of health inequality, whereas differences (D) and ratios (R) are simple measures. Also, while difference (D) ACI, and (PAR), are absolute measures, RCI, R and (PAF) are relative measures. Both the horizontal inequality index and the concentration curve were used to evaluate the wealth-related inequities. Using these diverse measures, the study can provide a nuanced and detailed picture of inequities in healthcare-seeking behavior, offering valuable insights for targeted interventions and policy formulation to improve child health outcomes in Ethiopia

Furthermore, determinants of inequity in healthcare seeking behavior were examined using a multilevel logistic regression analysis. The variables were classified as either community-level or individual-level factors after the data were examined at two different hierarchical levels. Survey year, region, and place of residence were community-level factors; wealth index, maternal and paternal education, age, occupation, religion, media use, child sex, birth order, antenatal care (ANC), and child delivery place were included under individual-level factors. These variables were selected based on their sensitivity on the utilization of child healthcare services.

For the two ordered measures, multilevel binary logistic regression was employed. Weighted samples were ranked from most disadvantaged (poorest and uneducated, ranked 0) to most advantaged (secondary education or higher and richest, ranked 1). Low or high coverage as well as urban/rural differences were used for the region. The following models have been fitted: random intercept with the fixed effects model, random intercept with the empty model, and random coefficient with a random intercept model. Using the likelihood ratio test (LRT) and deviation test, the heterogeneity of proportions between exposure variables in a multilevel analysis was examined in the first step. Lastly, R-Software version 4.3.2 was used to report the odds ratio (OR) with a 95% confidence interval (CI), and p-values less than 0.05 were regarded as statistically significant.

The probability that the response variables equal one is represented by pij. Pij =  P(yij =  1/xij) is the probability that the ith child received healthcare services in the jth variable.“

This is one way to represent the two-level logistic regression model:


$$logit(P_{ij})-\log\left[\frac{P_{ij}}{1-P_{ij}}\right]-\beta_{0}+\beta_{1}{\text{X}}_{\text{ij}}+U_{oj}$$

*U*_*oj*_ is a random quantity that has a mean of zero and a variance of σ2u, according to a normal distribution. One level 1 model and one level 2 model can be created from this one.


$$\begin{array}{l} logit(P_{ij})-\log\left[\frac{P_{ij}}{1-P_{ij}}\right]-\beta_{oj}+\beta_{1}{\text{X}}_{\text{ij}}\\ \left[\text{Model: level 1, random intercept model}\right] \end{array}$$

β−ojβo+Uoj[Model: level 2, empty model]

where $Pij-\frac{e^{\beta_{0}}+\sum_{h-1}^{k}\beta_{h}X_{hij}+U_{oj}}{1+e^{\beta_{0}}+\sum_{h-1}^{k}\beta_{h}X_{hij}+U_{oj}}$

Thus,


$$\text{logit}(P_{ij})-\log\left[\frac{P_{ij}}{1-P_{ij}}\right]-\beta_{oj}+\sum_{h-1}^{k}\beta_{h}{\text{X}}_{\text{ij}}+U_{oj}+\sum_{h-1}^{k}U_{hj}{\text{X}}_{\text{ij}}$$

In the multilevel logistic regression model with random intercept, the fixed part of the model is referred to as part one, and the random part as part two. Although they might be correlated within groups, the random variables or effects, U0j, U1j,..., Ukj, are thought to be independent between groups. Thus, the vector U0j, U1j,... Ukj’s components are distributed independently as a multivariate normal distribution with a zero mean vector, variances and covariances matrix Ω, and zero mean vector.

The intraclass correlation (ICC) is then provided by


$$\text{ICC}=\frac{\sigma_{\mu }^{2}}{\sigma_{\mu }^{2}+\sigma_{e}^{2}}$$

Where $\sigma_{\mu }^{2}$s the between group variance and $\sigma_{e}^{2}$ is the within group variance.

To examine the existence of multicollinearity between variables, the variance inflation factor (VIF) was computed in all cases. A VIF greater than 7.5 suggests the possibility of multicollinearity. Heteroscedasticity, where the variance of residuals is unequal across levels of an independent variable, can lead to inefficiency in estimates. To test for this, we performed a Breusch-Pagan test or White’s test for heteroscedasticity. If heteroscedasticity was detected, we might have used robust standard errors or employed generalized least squares (GLS) to account for heteroscedasticity, ensuring more reliable and efficient estimates. In case of sensitivity analyses are crucial to ensure the robustness of findings. For this we performed sensitivity analyses by re-running models with different assumptions or subgroups. For example, excluding certain regions, wealth quintiles, or variables, and reanalyzing the data could test the stability of the results. Another form of sensitivity analysis might involve testing different model specifications or including/excluding control variables to ensure that the core results regarding inequity in healthcare-seeking behavior remain consistent.

### Ethical consideration

The study used publicly available data from the Ethiopian Demographic and Health Surveys (EDHSs). All DHS surveys were authorized by ICF and an Institutional Review Board (IRB) in the corresponding nations to ensure that the protocol conforms with U.S. Department of Health and Human Services regulations for the protection of human subjects. The Institutional Review Board (IRB) of Addis Ababa University’s College of Health Sciences granted ethical approval.

## Result

### Socio-demographic characteristic of the study participants

Around 8,250 under-five children, who fulfilled the inclusion criteria were participated in this study. Nearly, 50.3% (4152) of them were males, and 49.7% (4098) of them were female participants. Regarding residence, 13.4% (1,102) of them came from urban area and 86.6% (7,148) of them were rural residences. The overall prevalence of symptoms of diarrhea disease was decreased from 26% in (2000) to 11.8% in (2016), symptoms of ARI from 24.4% in (2000) to 6.6% in (2016) and fever from 28.4 (2000) to 14.3% in (2016) among under- five children. While, healthcare seeking behavior for diarrheal, ARI and symptoms of fever was showed an increasing trend from 21.1% in (2000) to 44.1% in (2016), from 21.3% to 29.1%, and from 23.7% to 35.7% respectively (Table 1). There was a significant increase in healthcare seeking behavior for diarrhea over this period by 23% (Table 1).

**Table 1:** Trends of under- five children with diarrhea, Pneumonia and fever get treatment from health facility in Ethiopia.

Survey Year	Proportion of Diarrhea disease (%)	Percentage of children with Diarrhea Received ORS (%)	Proportion of ARI (%)	Percentage of children with ARI who receive treatment from health facility (%)	Proportion of fever(%)	Percentage of children with fever who receive treatment (%) CI
2000	23.6	21.1 (13.8, 24.5)	24.4	21.3(16.2, 37.3)	28.4	23.7 (16.8, 27.9)
2005	18	25.2(20.1, 45.3)	12.6	20.2(13.4, 38.2)	18.7	18.8(0.9, 28.9)
2011	13.4	35.1(27.3, 48.6)	7.0	29.1(25.2, 56.3)	17.1	25.8(18.8, 40.6)
2016	11.8	44.1 (43.3, 45)	6.6	31.6(23.2, 49.3)	14.3	35.7 (34.3, 35.7)

### Levels and trends of inequity, in health care seeking behavior for symptoms of diarrhea disease for under- five children in Ethiopia

The level and trend of healthcare seeking activity for diarrhea symptoms have increased over time in each survey year. Over the course of the year, the richest wealth quintile practiced a comparatively higher rate of healthcare seeking behavior and use of health facilities for symptoms of diarrhea than the poorest wealth quintile. In the richest groups, the percentage of people seeking healthcare was higher by 25 percentage points in 2000, 24.3 percentage points in 2005, 27 percentage points in 2011, and 14 percentage points in 2016. But, the trend and the point difference in healthcare seeking behavior were higher among the poor over the rich over the last sixteen years. Compared to children whose mothers had no formal education, a similar pattern was also seen in those whose mothers had completed secondary or higher education., Regarding the sex of the child, male children’s healthcare seeking behavior increased by 3 and 4.5 percentage points between 2005 and 2011. Children from urban areas were also more likely than those from rural areas to seek medical care in all survey waves. There was a variation in healthcare seeking behavior for child diarrhea symptoms across subnational regions. A more in-depth look between 2000 and 2016 indicates that among those sub-regions with a reasonable number of point estimates, treatment seeking across regions ranged from 12.9% to 55.8% for symptoms of diarrhea.

For instance, it was higher in the Addis Ababa administrative compared to other regions 45.5% in (2005) and 55.8% in (2016), while it was higher in Somalia and Gambela regions 50.5% and 45.3% in 2000 and in 2011 respectively (Table 2).

**Table 2:** Levels and trends of healthcare seeking behavior for symptoms of diarrhea disease for under- five children in Ethiopia, disaggregated across five inequality dimensions, 2000- 2016.

Dimension of inequalities	2000		2005		2011		2016	
	%(95%)CI	Popn	%(95%)CI	Popn	%(95%)CI	Popn	%(95%)CI	Popn
**Economic status**								
poorest	8.2(5.6,12)	20.9	13.5(9.7,18.40	21.7	18(13.3,23.9)	25.1	26.7(19.8,35)	20.7
poorer	8.9(6.1,12.7)	22.8	13.5(9.3,19.1)	23.5	22.4(15.9, 30.7)	20.3	27.7(19.4,37.9)	23.1
Middle	10.1(7.4,13.6)	22.2	23.1(16.6, 31.3)	24.0	27(20.5, 34.6)	20.1	32.424.5, 41.4)	21.8
Rich	12.7(9.6,16.6)	20.7	19.8(13.9, 27.4)	18.6	27.7(21.5, 34.9)	21.8	23.4(15.5,33.8)	20.6
Richest	33.2(24.7,43)	13.4	37.8(30.4,45.8)	12.2	45.3(35,56)	12.8	41.4(32.6, 50.8)	13.8
**Education**								
No Education	10.9(9, 13)	84.3	17(13.9,20.7)	79.3	23.5(19.9,27.4)	71.4	27.9(23.2,33.1)	62.5
Primary School	19.2(13.3,26.8)	12.0	28.4(21.9(35.8)	18.2	29.7(24,36.1)	25.6	31.6(25.3,38.9)	30.1
Secondary school +	42.9(26.9, 60.6)	3.7	51.6(32.9,69.8)	2.4	65.1(44,81.6)	3.0	35.1(23,49.4)	7.4
**Residence**								
Rural	10.3(8.8, 10.9)	92.5	18.6(15.6,21.9)	95.0	24.1(20.8,27.8)	89.4	28.3(24.4,32.5)	89.7
urban	47.3(35.1,59.8)	7.5	45.7(32,60.1)	5.0	44.6(33,56.8)	10.6	40.5(31.3,50.6)	10.3
**Sex**								
Female	12.8(10.5, 15.5)	47.9	18.4(14.9, 22.4)	49.4	23.8(19.5,28.8)	45.1	28.8(23.6,34.5)	47.1
Male	13.3(10.7,16.3)	52.1	21.4(17.6,25.8)	50.6	28.3(24.1, 33)	54.9	30.2(25.2,35.7)	52.9
**Subnational Region**								
**Adis Ababa**	40.1(23.1, 59.9)	0.8	45.3(31.1, 60.4)	1	43.4(25.9, 62.6)	1.4	55.8(40.9, 69.6)	1.4
**Afar**	23.6(11.3, 43)	0.7	8.8(2.1, 30.4	0.7	32.8(21.2, 46.9)	1	32.9(24.4, 42.6)	1.0
**Amhara**	9(6.6, 12.4)	21.3	19.9(14.5,26.8)	18.5	27.6(20.3, 36.4)	22.8	28.4(20.8, 37.2)	2.2
**Benishangul Gumz**	13.4(10.2, 17.5)	1.1	24.9(17.8, 33.7)	1.1	28.7(21.6, 36.9)	1.9	55.3(42.2, 67.7)	0.8
**Dire dewa**	27.9(18.1, 40.4)	0.3	31.3(19.4, 46.3)	0.2	42.8(33.4, 52.8)	0.2	51.1(35.1, 66.9)	0.4
**Gambela**	32.6(24.1, 42.3)	0.3	27.6(15.8, 43.5)	0.2	45.3(33.5, 57.9)	0.6	39.7(29,51.5)	0.3
**Harreri**	26.2(18.1, 36.2)	0.2	22(14.4, 33.6)	0.2	38.6(27, 51.8)	0.2	39.1(26.1,53.8)	0.2
**Oromia**	12.9(9.9, 16.7)	42.9	22.6(17, 29.4)	39	23.8(18.4,30.3)	35.7	22.5(16.9, 29.3)	39.7
**SNNPR**	13(9.5, 17.4)	26.6	15.9(12.1,20.6)	31.4	25.1(19.1, 32.1)	25.5	33.3(26, 41.6)	24.5
**Somalia**	50.5(28.3, 72.3)	1.1	15.8(7.8, 29.2)	2.9	30.6(20.4,43.1)	4.5	44.2(29,60.6)	2.3
**Tigry**	15.3(9.5, 23.8)	4.8	21.1(13.5, 31.5)	4.6	29.2(22.8,36.6)	6.3	43(33.2 53.4)	7.3

### Levels and trends of healthcare seeking behavior for symptoms of ARI and fever for under- five children in Ethiopia

Level of healthcare seeking behavior for symptoms of ARI and fever treatment was varied based on economic status, educational status, place of residence and subnational regions with in the country. In terms of economic status, the richest group’s health care seeking behavior symptoms of ARI and fever climbed between 2000 (39.3%) and 2011 (61.7%), but declined to 35.2% in 2011 and 37.6% in 2016.

In the poorest quintile, there was a decline from 25.8% in 2005 to 15.4% in 2011, followed by a rebound to 24.9% in 2016. Regarding maternal education, level of healthcare seeking behavior was higher among primary school sub-groups in all the four surveys compared to no education sub-groups (Table 3). The level of healthcare seeking behavior was higher among urban dwellers than among rural residents, and this difference persisted across all four rounds of the survey. The level of healthcare seeking behavior for pneumonia treatment was increased from (48.1% to 55.7%) in the urban area and from (19.3% to 27.4%) in rural area between 2000–2016. Regarding sex-based inequity in health care seeking behavior for pneumonia and fever treatment, the level shows insignificant differences between male and female children in all round surveys (Table 3). In another way, this study revealed that, there were huge variations in health care seeking behavior for both morbidities across subnational regions from 2000 to 2016. For example, it was as higher as 63.5% in Addis Ababa and lowest 13.2% in Tigray in 2000 and 44.3% in Afar and 23.1% in Oromia in 2016 (Table 3).

**Table 3:** Levels and trends of healthcare seeking behavior for symptoms of ARI and fever for under- five children in Ethiopia, disaggregated across five inequality dimensions: 2000–2016.

Dimension of inequalities	2000		2005		2011`		2016	
	%(95%)CI	Popn	%(95%)CI	Popn	%(95%)CI	Popn	%(95%)CI	Popn
**Economic status**								
poorest	11.5(9.5, 21.5)	**51**	25.9(15.9, 39.1)	**157**	15.5(9.8, 23.6)	**188**	25(13.1, 42.3)	**133**
poorer	18.5(11.3, 34.7)	**55**	14.2(7.3, 25.9)	**173**	25.2(16.2, 37)	**148**	25.7(16.8,37)	**172**
Middle	22.2(19.7, 29.5)	**66**	25(16.7, 35.7)	**219**	22.1(14.7, 31.8)	**197**	27.4(20,36.4)	**176**
Rich	20.3(12.6, 34.8)	**54**	17(11, 25.4)	**163**	33.2(22, 46.7)	**173**	36.8(24.9, 50.7)	**147**
Richest	39.3(29.7, 49.6)	**36**	35.2(23.1, 49.6)	**110**	61.7(45.1, 76)	**67**	37.6(21.6, 56.9)	**63**
**Education**								
No Education	**19.5(17.8, 56.7)**	**219**	**18(13.6, 23.4)**	**637**	**24.6(19.4, 30.8)**	**551**	**26(20, 33)**	**475**
Primary School	**24.4(23.6, 36.5)**	**32**	**35.3(25.3, 46.8)**	**165**	**27.7(19.5,37.8)**	**199**	**35.2(26.6, 44.9)**	**177**
Secondary school +	**49.1(38.9, 58.3)**	**10.**	**NA**	**20**	**NA**	**22**	**44.5(21.9,69.6)**	**38**
**Residence**								
Rural	**19.3(14.1, 29.2)**	**244**	**21.7(17.5, 26.7)**	**787**	**25(20.3, 30.4)**	**703**	**27.5(22.3,33.4)**	**643**
urban	**48.1(45.1, 67.1)**	**18**	**43(25,63.2)**	**37**	**46.9(24, 71.2)**	**69**	**55.7(35.4, 74.3)**	**48**
**Sex**								
Female	**20.2(16.3, 32.2)**	**128**	**22.4(16.6, 29.5)**	**402**	**28.7(22.1,36.3)**	**380**	**26.7(20.4,34.1)**	**342**
Male	**22.3(15.1, 28.7)**	**134**	**23(17.7, 29.3)**	**422**	**25.4(19.1, 32.9)**	**393**	**32.1(24.9,40.2)**	**349**
**Subnational Region**								
**Adis Ababa**	63.5(52.2, 76.3)	17	NA	**3.2**	NA	**7.2**	NA	**6.3**
**Afar**	31.1(23.5, 45.4)	24	NA	**3.5**	40.6(25.2, 58)	**6**	44.3(33.4, 55.7)	**4.5**
**Amhara**	14.9(0.78, 23.8)	606	17.5(9.8, 29.3)	**138**	29.4(19.6, 41.5)	**159**	29.1(19.7, 40.5)	**157**
**Benishangul Gumz**	31.5(23.4, 45.8)	32	24(12.2, 41.8)	**5.8**	42.9(33.1, 53.3)	**13.4**	NA	**2.1**
**Dire dewa**	37.8(25.1, 48.8)	5	NA	**0.6**	47.1(26.6, 68.7)	**2.5**	NA	**1.7**
**Gambela**	53(36.6, 78.4)	5	NA	**1.4**	52.5(41.3, 63.4)	**3.4**	NA	**0.9**
**Harreri**	41.5(19.9, 67.4)	4	41.8(19.8,67.7)	**1.2**	NA	**0.4**	NA	**0.2**
**Oromia**	22.7(8.9, 45.7)	1193	27.7(20.7, 35.9)	**374**	23.4(15.6, 33.6)	**328**	23.1(15.6, 32.80	**339**
**SNNPR**	23.9(20.8, 54.9)	508	20.1(13.6, 28.8)	**235**	31.6(23, 41.6)	**157**	43.2(32.7, 54.3)	**117**
**Somalia**	55(45.8, 76.3)	27		**15**	18.7(10.8, 30.4)	**29**	32.2(12.2, 61.9,)	**9.8**
**Tigry**	13.2(9.8, 26.3)	207	14.6(6.1, 31)	**44.6**	18.4(10.7, 29.8)	**66**	30.6(20.9, 42.4)	**53**

### Trends of inequities in healthcare seeking behavior for symptoms of diarrhea based on summary measures

The findings of the absolute and relative summary measures of inequity are shown in (Table 4). Significant disparities in healthcare seeking behavior by socioeconomic status, socio-demographic characteristics, and geographic location were found in Ethiopia between 2000 and 2016.

**Table 4:** Trends of inequity in healthcare seeking behavior for symptoms of diarrhea based on summary measures D,PAF, PAR, R, RCI and ACI, (2000-2016).

Dimension of inequality	Measure of inequality	2000	2005	2011	2016
		% (95% CI)	% (95% CI)	% (95% CI)	% (95% CI)
Economic status					
	D	25(15.2, 34.7)	24.4(15.5,33.2)	27.3(15.4, 39.2)	14.6(2.7, 26.5)
	PAF	154.1(137.2, 170.9)	89.8(74.1, 105.4)	72.1(58.6, 85.6)	40.1(23.4, 56.7)
	PAR	20.1(17.9, 22.3)	17.9(14.8, 21)	19(15.4, 22.5)	11.8(6.9, 16.8)
	R	4(2.5, 6.5)	2.8(1.9, 4.1)	2.5(1.7, 3.7)	1.5(1.1, 2.2)
	RCI	5.4(3.7, 8)	18.1(15.6, 20.6)	15.5(13.7,17.4)	4.8(4.2, 5.4)
	ACI	21.5(16.6, 26.4)	3.6(2.2, 5.0)	4.1(2.3,5.9)	1.4(-0.7, 3.5)
Education					
	D	32.1(14.5, 49.6)	34.6(15, 54.1)	41.7(21.7, 61.6)	7.2(-7.1, 21.5)
	PAF	228.9 (223.5, 234.2)	158.9(153.6, 164.2)	147.7(142, 153.3)	18.8(12, 25.5)
	PAR	29.9 (29.2, 30.6)	31.7(30.6, 32.7)	38.8(37.4, 40.3)	5.5(3.5, 7.5)
	R	4(2.5,6.2)	3 (2, 2.6)	2.8(2, 3.9)	1.3(0.8, 1.9)
	RCI	6(3.8,9.3)	12.1(10.3,13.9)	8.7(7.7, 9.7)	3.8(3.3, 4.3)
	ACI	23.8(17.4, 30.2)	2.4(1.2, 3.6)	2.3(0.9, 3.7)	-1.1(-0.7, 3.0)
Residence					
	D	37(24.4, 49.6)	27.2 (12.5, 41.9)	20.5(7.8, 33.1)	12.3(1.7, 22.8)
	PAF	262.1(257.4, 266.8)	129.6(126.7, 132.5)	69.5(66.1, 73)	37.3(34.1, 40.5)
	PAR	34.2(33.6, 34.8)	25.8(25.2, 26.4)	18.3(17.4, 19.2)	11(10.1, 12)
	R	4.6(3.4, 6.2)	2.5(1.7, 3.5)	1.8(1.4, 2.5)	1.4(1.1, 1.9)
	RCI	NA	NA	NA	NA
	ACI		NA	NA	NA
Sex					
	D	0.5(-4.2, 3.3)	-3(-8.6, 2.5)	-4.5(-11, 2)	-1.4(-9, 6.2)
	PAF	0(-9.6, 9.6)	0(-9.1, 9.1)	0(-7.7, 7.7)	0(-8.2, 8.2)
	PAR	0(-1.3, 1.3)	0(-1.8, 1.8)	0(-2, 2)	0(-2.4, 2.4)
	R	1(0.7, 1.3)	0.9(0.6, 1.1)	0.8(0.7, 1.1)	1(0.7, 1.2)
	RCI	NA	NA	NA	NA
	ACI	NA	NA	NA	NA
Subnational					
	D	41.5(17.7, 65.3)	36.5(17.2, 55.80	21.5(7.9, 35.1)	33.2917.3, 49.2)
	PAF	286.8(269.6, 304)	127.5(6.2, 248.8)	72.2(60.9, 83.5)	88.9(78.6, 99.20)
	PAR	37.5(35.2, 39.7)	25.4(1.2, 49.6)	19(16, 22)	26.2(23.2, 29.3)
	R	5.6(3.2, 9.8)	5.1(1.3, 21)	1.9(1.3,2.7)	2.5(1.7, 3.6)
	RCI	NA	NA	NA	NA
	ACI	NA	NA	NA	NA

**Fig 1:**
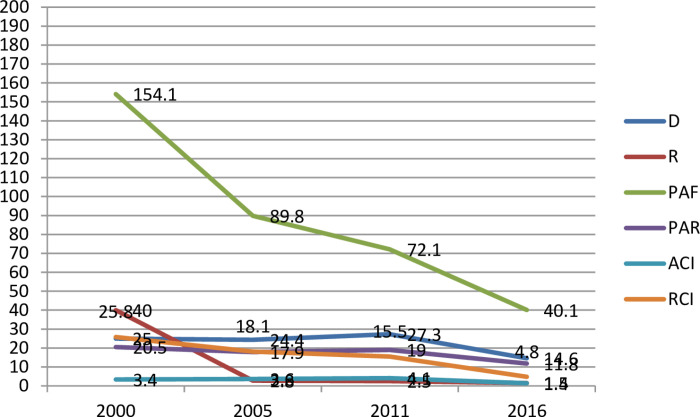
Wealth index related inequity for symptoms of diarrhea treatment.

In terms of wealth disparity, all five surveys revealed pro-rich inequity in the healthcare seeking behavior for diarrheal symptoms. From 2000 to 2016, the absolute measures of inequity D and R demonstrated persistent wealth-related inequity. In addition, the results showed that if absolute and relative wealth-driven inequity had not occurred, the PAR and PAF measures in 2016 may have improved by almost 11.8 and 40.1 percentage points, respectively, suggesting potential for improvement in the healthcare seeking behavior (Fig 1). Both absolute and relative maternal education measures revealed a substantial difference in healthcare seeking behavior for symptoms of diarrhea. The fact that the inequity trend did not always go up or down between surveys, suggests that the inequity by maternal education varied amongst survey years. More specifically, according to Table 4, RCI-related inequity grew by 5.0 percentage points between 2000 and 2005, fell by 3.4 percentage points between 2005 and 2011, and then decreased by 4.9 percentage points in 2016. Between surveys, there was a consistent decline in inequity related to place of residence. The basic measures of inequity R and D show that between 2000 and 2016, there was a decrease in the disparity between the rural and urban subgroups. In another way, the relative or absolute measures of sex-related inequality in healthcare seeking behavior for diarrheal symptoms did not change during the survey. In terms of regional trends, both simple and complex measures of inequity revealed notable variations between the regions, but overall there was a steady downward trend in disparities. Regional variations in PAF were observed in all survey years; the percentage point differences were 286.8, 127.5, 72.2, and 88.9 in 2000, 2005, 2011, and 2016, respectively. (Table 4).

### Trends of inequities in healthcare seeking behavior for symptoms of ARI and fever based on summary measure of inequity

This study included both relative (R, RCI, PAF) and absolute (D, RII, PAR) measures of inequity (Table 5). The results of this summary measure of inequity indicate that in 2011, there was pro-rich inequity in healthcare seeking behavior for symptoms of fever and ARI. However, in 2005 and 2016, the absolute measures of inequity (D & R) did not show any economic related inequity in in healthcare seeking behavior for pneumonia and fever. In another way, the wealthiest families were found to be somewhat more likely than the poorest families to seek treatment for ARI, according to the complex measures (RCI, PAR, and PAF).

**Table 5:** Trends of inequity in healthcare seeking behavior for symptoms of ARI and fever based on summary measures of inequity from 0-2016).

Dimension of inequality	Measure of inequality	2000	2005	2011	2016
		% (95% CI)	% (95% CI)	% (95% CI)	% (95% CI)
Economic status					
	D	27.8(19.1, 34.8)	9.4(-8.3, 27.0)	46.2(28.9, 63.6)	12.7(-10.8,36.1)
	PAF	87.2(56.1, 98.4)	55.2(28.2, 82.3)	128.6(110.7, 146.4)	27.8(4.8, 50.8)
	PAR	45.7(34.8, 64.2)	12.5(6.4, 18.7)	34.7(29.9,39.5)	8.2(1.4, 15)
	R	3.4(1.8, 4.9)	1.4(0.8, 2.4)	4.0(2.4, 6.7)	1.5(0.7, 3.2)
	RCI	18.9(12.6, 27.2)	5.0(4.0, 6.0)	21.0(17.4, 24.5)	8.8(7.2, 10.5)
	ACI	8.9(3.8, 24.9)	1.1(-1.5, 3.7)	5.7(3.1, 8.2)	2.6(-0.8, 6.0)
Education					
	D	29.6(23.8, 45.9)	NA	NA	18.4(-8.3, 45.0)
	PAF	87(45.6, 96.9)	NA	NA	51.0(42.9, 59.1)
	PAR	32(23.8, 54.9)	NA	NA	15.0(1269, 17.4)
	R	2.5(1.6, 5.9)	NA	NA	1.7(0.9, 3.2)
	RCI	12.8(8.9, 23.5)	NA	NA	8.3(6.8, 9.7)
	ACI	6.9(3.8, 9.7)	NA	NA	2.4(0.0, 4.9)
Residence					
	D	28.8(12.7, 43.9)	21.3(1.0, 41.6)	21.8(-4.1, 47.8)	28.3(7.1, 49.4)
	PAF	107(68.8, 156)	89.7(86.3, 93.1)	73.6(69.3,78.0)	89.4(85.5, 93.2)
	PAR	34.6(24.8, 68.2)	20.4(19.6, 21.1)	19.9(18.7, 21.1)	26.3(25.2, 27.4)
	R	2.5(1.6, 6.2)	2.0(1.2, 3.3)	1.9(1.0,3.3)	2(1.3, 3.1)
	RCI	NA	NA	NA	NA
	ACI	NA	NA	NA	NA
Sex					
	D	2.1(1.9, 6.2)	-0.5(-9.1, 8.0)	3.4(-6.5, 13.2)	-5.4(-15.6, 4.9)
	PAF	0.06(-23, 34)	0.0(-12.3, 12.3)	6.3(-5.1, 17.7)	0.0(-11.4, 11.4)
	PAR	0.0(-7, 6.9)	0.0(-2.8, 2.8)	1.7(-1.4, 4.5)	0.0(-3.4, 3.4)
	R	1.1(0.8, 5.3)	1.0(0.7, 1.4)	1.1(0.8,1.6)	0.8(0.6, 1.2)
	RCI	NA	NA	NA	NA
	ACI	NA	NA	NA	NA
Subnational					
	D	50.3(28.9, 68.9)	NA	NA	NA
	PAF	89.7(67.8, 98.6)	NA	NA	NA
	PAR	12.8(5.8, 24.7)	NA	NA	NA
	R	4.8(2.8, 12.8)	NA	NA	NA

This study compared differences in healthcare seeking behavior based on education using data from only two years, 2000 and 2016. Accordingly, households with more educated mothers were more likely than households with less educated mothers to seek treatment for suspected symptoms of ARI for their child. This is consistent with both absolute and relative complex measures of D and RCI indicating inequity. Furthermore, according to Table 5, absolute measures of inequality (D) show a consistent upward trend in residence-based inequity over time. With respect to the disparity in sex-related health care seeking behavior that occurred between 2000 and 2016, no measure of inequity revealed any indication of sex inequality in the healthcare seeking behavior for symptoms of ARI and fever among under –five children in Ethiopia.

**Fig 2:**
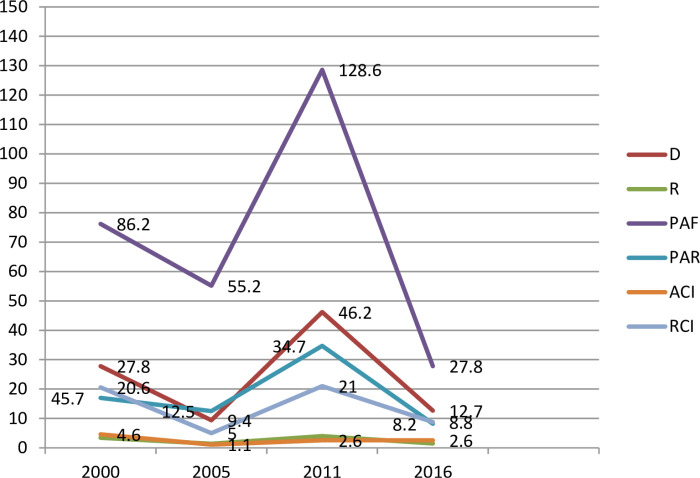
Wealth index related inequity for symptoms of ARI and fever treatment.

**Fig 3:**
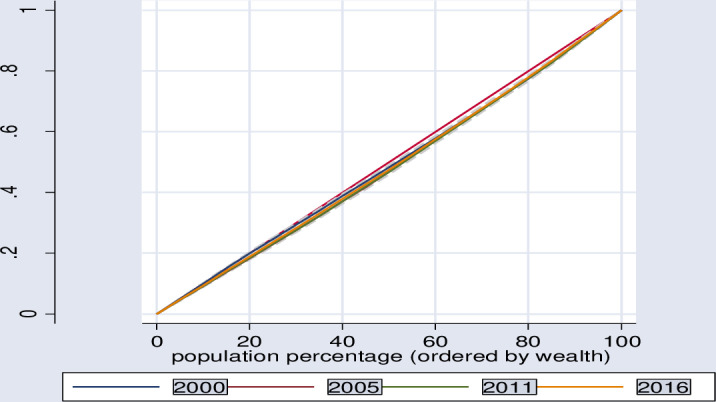
Lorenz curve for trend of inequity in health care seeking behavior for symptoms of diarrhea.

**Fig 4:**
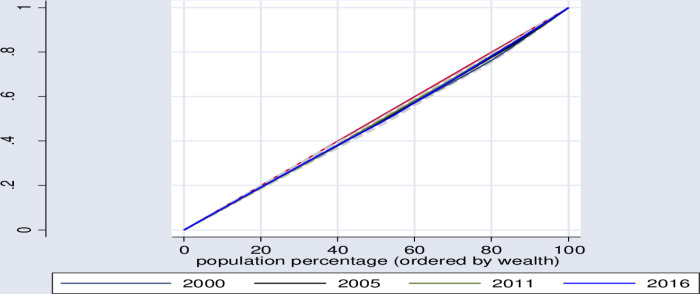
Lorenz curve for trend of inequity in health care seeking behavior for symptoms of ARI.

**Fig 5:**
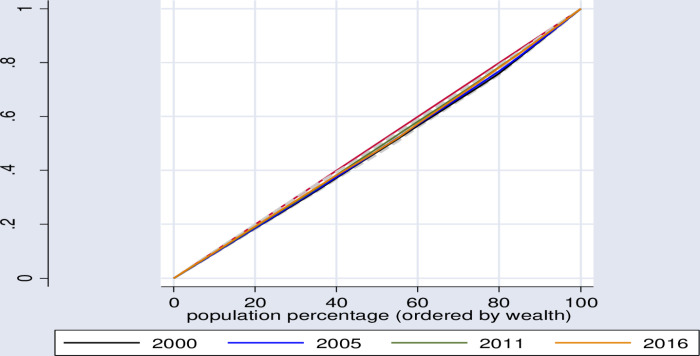
Lorenz curve for trends of inequity in health care seeking behavior for symptoms of fever.

In a similar manner, using the wealth index, Lorenz curve and concentration index show that, significant inequities in healthcare seeking behavior distribution was found among poor segment of the population sub-groups in Ethiopia (Table 6, Figs 2–5).

**Table 6:** Concentration Index of common childhood illness.

	Diarrhea	ARI	Fever
Year	Concentration index with 95%CI	concentration index with 95% CI	concentration index with 95% CI
2000	0.942(0.93, 0.946)	0.941(0.937, 0.945)	0.94(0.938, 0.943)
2005	0.943(0.938, 0.947	0.940(0.935, 0.946)	0.941(0.935, 0.946)
2011	0.941(0.939, 0.944)	0.942(0.938, 0.946)	0.944(0.938, 0.950)
2016	0.944(0.941, 0.947)	0.944(0.938, 0.947)	0.945 (0.939, 0.951)

### Multilevel analysis of inequity, in healthcare seeking behavior for diarrhea, fever and symptoms of ARI

The study utilized multilevel binary logistic regression to determine the factors that contribute to inequities in healthcare seeking behavior for symptoms of fever, diarrhea, and ARI. The study reveals that certain individual-level factors, such as the household wealth index, parental education, media use, ANC utilization, place of delivery, birth order, and rapid birthing, show statistically significant associations with inequities in healthcare seeking behavior for symptoms of fever, diarrhea, and ARI. Among the community-level variables that were looked at, region, survey year, and place of residence were found to be statistically significantly linked to inequities in health care seeking behavior for symptoms such as fever, diarrhea, and ARI.

Compared to respondents from the poorest households, those from the richest wealth status category had 1.6 times higher odds of seeking healthcare for symptoms of fever, diarrhea, and ARI (OR: 1.6, 95% CI: 1.12–2.30). At the same time respondents who had exposure to media had 1.27 times more health care seeking behavior for diarrhea, fever and symptoms of ARI (OR: 1.27,95%CI: 1.05–1.53). Moreover mothers who have had four or more ANC follow up had 1.99 times more health care seeking behavior for diarrhea, fever and symptoms of ARI (OR: 1.99, 95% CI:1.63–2.43). Furthermore, mothers who gave birth in a medical facility were 1.79 times (OR: 1.79, 95% CI: 1.33–2.41) more likely than their counterparts to seek medical care for symptoms of fever, diarrhea, and ARI. Children of mothers who lived in rural areas had a 50% lower likelihood of seeking healthcare for symptoms such as fever, diarrhea, and ARI compared to children of mothers who lived in urban areas (OR: 0.50, 95% CI: 0.32–0.80). After keeping other variables constant, the likelihood that a child in the Addis Ababa region will seek medical care for symptoms such as fever, diarrhea, and ARI symptoms is five times higher than that of a child in the Afar region, (OR: 5.0, 95% CI: 2.28–11.15) (Tables 7–12).

**Table 10:** Factors contributing for healthcare seeking behavior for symptoms of ARI and fever in Ethiopia.

Predictors		Null Model	Model II AOR(95% CI)	Model III AOR(95% CI)
**Individual-level variables**				
**Age of respondent**	Years	–	0.98(0.97–1.00)	0.98(0.96–1.00)*
**Wealth**	Poorest	–	1	1
	Poorer	–	1.19(0.90–1.56)	1.17(0.88–1.54)
	Middle	–	1.45(1.10–1.91)**	1.44(1.10–1.90)**
	Richer	–	1.18(0.88–1.59)	1.19(0.89–1.60)
	Richest	–	1.90(1.31–2.75)**	1.61(1.09–2.39)*
**Education**	No Education	–	1	1
	Primary	–	1.21(0.97–1.51)	1.14(0.92–1.41)
	Secondary/Higher	–	2.06(1.27–3.35)**	1.86(1.20–2.90)**
**Gender**	Male	–	1	1
	Female	–	0.96(0.82–1.13)	0.94(0.80–1.10)
**Birth order**	First	–	1	1
	Second	–	0.90(0.68–1.19)	0.91(0.69–1.19)
	Third	–	1.24(0.92–1.67)	1.26(0.95–1.68)
	Forth or above	–	0.92(0.67–1.26)	0.96(0.71–1.29)
**ANC**	One	–	1	1
	Two	–	1.15(0.81–1.62)	1.17(0.83–1.64)
	Three	–	1.54(1.10–2.15)*	1.49(1.08–2.06)*
	Four or more	–	1.92(1.55–2.38)**	1.68(1.36–2.08)**
**Place of delivery**	Home	–	1	1
	Health facility	–	1.81(1.33–2.46)**	1.51(1.12–2.04)**
**Media**	No	–	1	1
	Yes	–	1.28(1.04–1.57)*	1.19(0.98–1.45)
**Religion**	Orthodox	–	1	1
	Protestant	–	1.27(0.97–1.68)	0.90(0.66–1.22)
	Muslim	–	1.26(0.97–1.63)	1.07(0.81–1.41)
	Other	–	0.95(0.56–1.61)	0.74(0.43–1.28)
**Occupation**	No Job	–	1	1
	Has Job	–	1.04(0.87–1.24)	1.10(0.93–1.31)
**Rapid Breezing**	No	–	1	1
	Yes	–	1.58(1.31–1.92)**	1.67(1.38–2.01)**
**Partner education**	No education	–	1	1
	Primary	–	0.84(0.69–1.02)	0.77(0.64–0.93)**
	Secondary/Higher	–	1.11(0.78–1.57)	1.13(0.81–1.56)
**Community level variables**				
**Region**	Tigray	–	–	1
	Affar	–	–	0.99(0.33–2.91)
	Amhara	–	–	1.34(0.85–2.12)
	Oromiya	–	–	2.11(1.36–3.27)**
	Somali	–	–	1.13(0.51–2.51)
	Ben Gumz	–	–	2.07(0.88–4.87)
	SNNP	–	–	2.82(1.75–4.53)**
	Gambella	–	–	2.43(0.61–9.74)
	Harari	–	–	2.80(0.45–17.59)
	Addis Ababa	–	–	5.04(2.28–11.15)**
	Dire Dawa	–	–	1.89(0.43–8.27)
**Residence**	Urban	–	–	1
	Rural	–	–	0.50(0.32–0.80)**
**Survey Year**	2000	–	–	1
	2005	–	–	1.33(0.95–1.88)
	2011	–	–	2.51(1.81–3.47)**
	2016	–	–	2.38(1.66–3.42)**
	Intercept	0.25(0.22–0.27)**	0.08(0.05–0.14) **	0.07(0.03–0.15)**

Note: *  p-value <  0.05, ** p-value <  0.01, 1 reference category

**Table 11:** Measures of variations and model fit statistics in cough treatment among children in EDHS.

	Null Model	Model II	Model III
**Random effect result**			
ICC	29%	35%	34%
**Model fit statistics**			
Log-likelihood	-4006.878	-2603.059	-2570.417
AIC	8017.755	5256.117	5218.834
DIC	8013.755	5206.117	5140.834

**Table 12:** Measure of multicollinearity healthcare seeking behavior for symptoms of ARI and fever in Ethiopia.

Variable	VIF
Wealth	3.41
Education	2.31
Place of residence	3.67
Sex of a child	1.89
region	2.43
Place of delivery	3.65
ANC use	3.76
Media use	2.56
Rapid breezing	3.55
Survey year	3.54

**Table 7:** Factors contributing for healthcare seeking behavior for diarrhea disease in Ethiopia.

Predictors		Null Model	Model II AOR(95% CI)	Model III AOR(95% CI)
**Individual-level variables**				
**Age of respondent**	Years	–	0.99(0.98–1.01)	0.99(0.98–1.01)
**Wealth**	Poorest	–	1	1
	Poorer	–	0.92(0.73–1.16)	0.92(0.74–1.16)
	Middle	–	1.12(0.88–1.44)	1.13(0.89–1.43)
	Richer	–	0.97(0.74–1.27)	1.00(0.77–1.29)
	Richest	–	1.54(1.08–2.21)*	1.60(1.12–2.30)*
**Education**	No Education	–	1	1
	Primary	–	1.38(1.11–1.71)**	1.30(1.06–1.59)*
	Secondary/Higher	–	1.27(0.74–2.19)	1.32(0.82–2.15)
**Sex**	Male	–	1	1
	Female	–	0.80(0.68–0.93)**	0.79(0.69–0.92)**
**Birth order**	First	–	1	1
	Second	–	0.94(0.71–1.25)	0.93(0.71–1.21)
	Third	–	1.16(0.87–1.55)	1.13(0.86–1.48)
	Forth or above	–	1.00(0.74–1.35)	1.00(0.75–1.33)
**ANC**	One	–	1	1
	Two	–	1.03(0.74–1.45)	1.03(0.74–1.42)
	Three	–	2.16(1.58–2.95)**	1.99(1.49–2.67)**
	Four or more	–	2.28(1.86–2.80)**	1.99(1.63–2.43)**
**Place of delivery**	Home	–	1	1
	Health facility	–	2.26(1.65–3.10)**	1.79(1.33–2.41)**
**Media**	No	–	1	1
	Yes	–	1.30(1.07–1.59)**	1.27(1.05–1.53)*
**Religion**	Orthodox	–	1	1
	Protestant	–	1.01(0.79–1.30)	0.94(0.70–1.25)
	Muslim	–	1.23(0.96–1.57)	1.23(0.95–1.61)
	Other	–	1.46(0.94–2.25)	1.40(0.90–2.18)
**Occupation**	No Job	–	1	1
	Has Job	–	1.13(0.95–1.34)	1.14(0.97–1.34)
**Partner education**	No education		1	1
	Primary		1.04(0.87–1.24)	1.00(0.84–1.19)
	Seconday/Higher		1.21(0.86–1.72)	1.21(0.88–1.67)
**Community level variables**				
**Region**	Tigray	–	–	1
	Affar	–	–	0.48(0.18–1.30)
	Amhara	–	–	1.25(0.82–1.89)
	Oromiya	–	–	1.17(0.76–1.80)
	Somali	–	–	0.95(0.48–1.89)
	Ben Gumz	–	–	1.34(0.66–2.73)
	SNNP	–	–	1.32(0.85–2.07)
	Gambella	–	–	1.30(0.39–4.28)
	Harari	–	–	1.11(0.25–4.98)
	Addis Ababa	–	–	0.44(0.19–1.00)
	Dire Dawa	–	–	0.95(0.25–3.58)
**Residence**	Urban	–	–	1
	Rural	–	–	0.67(0.43–1.03)
**Survey Year**	2000	–	–	1
	2005	–	–	1.01(0.76–1.35)
	2011	–	–	1.67(1.25–2.24)**
	2016	–	–	2.31(1.67–3.18)**
	Intercept	0.47(0.44–0.51)**	0.20 (0.12–0.33) **	0.22(0.11–0.46)**

Note: *  p-value <  0.05, ** p-value <  0.01, 1 reference category

**Table 8:** Measures of variations and model fit statistics in diarrhea treatment among children in EDHS.

	Null Model	Model II	Model III
**Random effect result**			
ICC	23%	29%	28%
**Model fit statistics**			
Log-likelihood	-3956.996	-2750.968	-2731.568
AIC	7917.993	5549.935	5539.136
DIC	7913.993	5501.932	5463.136

ICC: Intra class correlation, AIC: Akaike Information Criteria, DIC: Deviance Information Criterion, A higher log-likelihood value indicates a better model fit., A lower AIC and DIC value indicates a better model fit.

**Table 9:** Measure of multicollinarity for healthcare seeking behavior for diarrhea disease in Ethiopia.

Variable	VIF
Wealth	4.12
Education	3.54
Place of residence	4.5
Sex of a child	2.71
region	1.3
Place of delivery	4.3
ANC use	4.1
Media use	3.8
Survey year	2.3

In this study, we calculated the Variance Inflation Factors (VIFs) for the independent variables included in the multilevel model. All VIF values were below the threshold of 7.5, indicating no severe multicollinearity in the data. This suggests that the regression coefficients are stable and the relationships between predictors in the analysis are appropriately specified. The results from the multilevel analysis show that healthcare-seeking behavior in Ethiopia has improved over time, but significant inequities persist based on wealth, education, ANC service utilization, media use, place of delivery and survey year.

Since all VIF values were below the critical threshold, no further action was required to address multicollinearity. The absence of significant multicollinearity suggests that the multilevel model is well-specified and the estimates of health-seeking behavior trends and inequities are reliable.

## Discussion

The objective of this study was to asses patterns of inequities in healthcare seeking behavior for childhood illness such as; diarrhea, fever, and ARI symptoms in Ethiopia. In this study, overall levels of treatment seeking behavior for symptoms of diarrhea, fever and ARI varied across socio-economic, demographic and sub-regions, by illness symptoms, and among surveys carried out between 2000 and 2016. The study revealed that the overall prevalence of symptoms of diarrhea disease was decreased from 26% in 2000 to 11.8% in 2016, symptoms of ARI from 24.4% in 2000 to 6.6% in 2016 and fever from 28.4 to 14.3% in 2016. While, healthcare seeking behavior for diarrheal, ARI and symptoms of fever was showed an increasing trend from 21.1% in 2000 to 44.1% in 2016, from 21.3%)in 2000 to 29.1% in 2016 and from 23.7% in 2000 to 35.7% in 2016 respectively. Despite this rise, significant inequities were persisted by wealth index, maternal education, sex of the child, place of residence, and region of the country. For example, pro-rich disparity in healthcare seeking behavior was present in all four surveys based on summary measures of inequity. With the exception of 2011, the wealth index relative inequity (D) decreased steadily from survey to survey. But, the relative inequity measure RCI was 5.4% in 2000, 18.1% in 2011 and 15.5% in 2016, revealing incremental pro-rich inequities in healthcare seeking behavior for symptoms of diarrhea based on economic status. Similarly, the absolute complex measure PAR declined from 34.7% in 2011 to 8.2 in 2016 for health care seeking behavior for symptoms of ARI. Indicating the possibility for improvement, if the average population sub-groups had the same level of health as the reference groups. The possible reasons could be household wealth status was consistently related with the three outcome variables, namely, healthcare seeking behaviors for childhood diarrhea, ARI, and fever. In-line with this, compared to mothers from the poorest homes, mothers from the richest, rich, and middle-income households were more likely to seek medical care. This result is in line with research from Ethiopia and Uganda that showed those in the lowest socioeconomic class experienced longer delays in seeking care for childhood fever than those in the highest socioeconomic class [39–41]. Several studies have found that the most important factor influencing the decision to medical care is one’s economic status [42–44]. The inequity caused by the households’ wealth status can be explained as follows: Wealth, as a proxy for income, has a beneficial impact on health care utilization, whereas a lack of financial resources can create hurdles to access. Even if health care is provided at a lower user charge in Ethiopia’s rural areas, extra costs such as transportation and prescription strain a household’s ability to pay; hence, the poorest portion of the population is unable to utilize health services. In other way, countries like Ghana and Rwanda, which have implemented more robust healthcare insurance systems and decentralized healthcare services, have seen improvements in healthcare equity. Ghana’s National Health Insurance Scheme (NHIS) and Rwanda’s Mutuelles de Santé have been effective in reducing the financial barriers to healthcare access, particularly among poorer households (1). This could be compared to Ethiopia, where such systems are less established. To address these healthcare disparities in Ethiopia, the government of Ethiopia should strengthening the Community-Based Health Insurance (CBHI) program by expanding coverage to rural and low-income populations can reduce financial barriers, drawing lessons from successful models like Ghana’s NHIS and Rwanda’s Mutuelles de Santé and mitigate economic disparities, the government should introduce targeted healthcare subsidies covering transportation costs, prescription fees, and other out-of-pocket expenses. Additionally, promoting maternal education through literacy programs and health awareness campaigns can empower mothers to recognize and respond promptly to childhood illnesses, with intensified media-based health promotion in rural areas.

In this study, even though the healthcare seeking behavior show higher among the higher wealth index group compared to the poorest group, the health care seeking behavior is increased by three fold for symptoms of diarrhea and by nearly by two folds for symptoms of ARI among the poor groups during the mentioned periods 8.2% in 2000 and 26.7% in 2016 and 11.5% in 2000 and 25% in 2016 respectively. This may be because the Ethiopian government placed a strong focus on meeting the health care needs of the poor. Initiatives like offering free health insurance and covering service costs may help the poor and avoid financial difficulties, which may encourage them to seek medical care.

Our study finding showed that variation in healthcare seeking behavior for children with diarrhea, fever, and ARI symptoms based on maternal and paternal educational status. For instance, in 2016, the absolute difference in health seeking behavior for symptoms of diarrhea and ARI between a mother who attend secondary school and no education is 7.2% and 18.4% respectively. This finding is consistent with several studies in Sub-Saharan Africa, Uganda, Nairobi, and India have found that low maternal and parental education is a predisposing factor to low health care seeking behavior for childhood diarrheal diseases [45–49]. This finding is also corroborated by research which suggests that poor maternal educational level in Ethiopia is connected with low maternal health care utilization [49–52]. Besides, Ethiopian women with higher education have a larger chance of using health services than women without education [13]. It is reasonable to believe that education is linked to improved awareness of illnesses, symptoms, and service availability. Moreover, educational attainment is a reliable indicator of socioeconomic position because it increases the ability to cope with the numerous costs involved [53]. Furthermore, education is likely to improve female autonomy, so that mothers have stronger confidence and capacities to make decisions regarding their children’s health [54].

In our study, inequities pertaining to residence steadily declined. The data indicated that, from 2000 to 2016, there was a decrease in the gap between the rural and urban subgroups, with the exception of ARI and fever in 2016.

In 2016 saw a clear disparity in the number of urban patients seeking treatment for pneumonia compared to those living in rural areas. There have been conflicting results regarding living in rural and urban areas up to this point; some studies suggest that their poverty and lack of access to healthcare make them more disadvantageous [55]. While others have not established, a substantial relationship between rural-urban residence and health care seeking behavior [56]. Timely recognition of symptoms of childhood illnesses is the first step toward seeking remedies; however, research have suggested that this does not generally take place in underserved locations around the world, such as rural or urban slums [57]. As a result, there is a need to raise community awareness about the need of early detection and subsequent referral to suitable health care services.

With respect to disparity in sex-related health care seeking behavior that occurred between 2000 and 2016, no measure of inequity revealed any indication of sex inequality in the health care seeking behavior for children with diarrhea, fever, and ARI symptoms. This is in line with the study done elsewhere in Africa and Ethiopia [58,59]. This could be due to cultural shifts and increasing public awareness on child health: While historically some cultures may have favored boys over girls in terms of healthcare access, over time, there has been a growing recognition of the value of gender equality [60]. In Ethiopia, the absence of sex-related disparities in healthcare-seeking behavior for children with diarrhea, fever, and ARI symptoms between 2000 and 2016 suggests progress toward gender equality in healthcare access.

To sustain and further strengthen this achievement, policymakers should continue implementing gender-sensitive health policies that promote equitable healthcare access for all children. Expanding community-based health education programs focused on child health and gender equality can reinforce cultural norms that value equal treatment for boys and girls.

Regarding regional patterns, there was a variation in health care seeking behavior for child diarrhea, symptoms of pneumonia and fever across subnational regions. A more in-depth look between 2000 and 2016 indicates that among those sub-regions with a reasonable number of point estimates, treatment seeking across regions ranged from 12.9% to 55.8% and 13.2% to 52.5% for symptoms of diarrhea and pneumonia respectively.

For instance, it was higher in the Addis Ababa administrative compared to other regions 45.5% in (2005) and 55.8% in (2016), while it was higher in Somalia and Gambela regions 50.5% in 2000 and 45.3% in 2011 respectively for diarrheal disease. Although there were notable regional variations in the simple and complex measures of inequity, the overall trend was showed a steady decrease in inequities. Each of the four survey years showed significant PAF in the regions: the differences in percentage points between 2000, 2005, 2011, and 2016 were 286.8, 127.5, 72.2, and 88.9. Potential explanations for the continued high geographic disparities include the scattered and isolated pastoral nomad settlements in the Afar, Somali, and Gambella regions, as well as the mountainous terrain of the central and northern highlands, which makes travel challenging. Multilevel binary logistic regression revealed that inequities in the healthcare seeking behavior for diarrhea, fever, and ARI symptoms were statistically associated with the household wealth index, parental education, media use, ANC utilization, place of delivery, birth order, region, survey year, and place of residence. For instance, compared to respondents from the poorest households, those from the wealthiest wealth status category had 1.6 times higher odds of seeking medical care for symptoms such as fever, diarrhea. Previous research [61,62] has shown that women living in poorer households are less likely to seek treatment for their sick child than women living in more affluent households. Across all sub-regions, women in the two lowest (poorest) quintiles of the household wealth index were consistently less likely to get their child treated when they had diarrhea, fever, and ARI symptoms. This is probably caused by several reasons, such as: (1) insufficient income to cover user fees or medication costs; (2) transportation expenses to and from the medical facility; and (3) time missed from work or farming in order to take the child for treatment [63–65]. This highlights the need for policymakers to invest in expanding health communication campaigns through mass media platforms such as radio, television, and social media, particularly targeting rural and underserved populations. Expanding access to affordable media infrastructure in rural areas, coupled with culturally tailored health campaigns, can bridge information gaps, reduce healthcare inequities, and ultimately improve health outcomes for under-five children in Ethiopia and similar contexts.

Our findings indicate that rural residents face significantly higher odds of childhood diarrhea compared to their urban counterparts. The likelihood of seeking healthcare for symptoms of acute respiratory infection (ARI) and fever was 50% lower among children of mothers residing in rural areas compared to those in urban settings. This disparity may be attributed to limited access to healthcare facilities, lower maternal education levels, and reduced healthcare awareness in rural regions. Similar studies have confirmed that children in rural households, often situated far from medical facilities, are consistently less likely to receive timely treatment for illnesses [30,66,67].

Additionally, our study revealed that female children were 21% less likely to be taken to a health facility when experiencing diarrhea, highlighting a potential gender bias in healthcare-seeking behavior. This finding contrasts with previous research, which reported no association between a child’s sex and the likelihood of being taken to a health facility for treatment, regardless of illness symptoms [38,66,67]. The observed gender disparity in our study may reflect persistent cultural norms and gender-based inequalities in healthcare access in certain communities. Addressing these inequities requires targeted interventions focusing on rural health infrastructure, community education, and gender-sensitive health policies.

### Strength and limitation of the study

Although this study identified trends and critical factors promoting equity in health care seeking behavior in childhood morbidities, it has some limitations. First, we used cross-sectional data, which may restrict our conclusions about the causal relationship between predictor and outcome factors. Second, the analysis relied on self-reported childhood morbidity from diarrhea, fever, and symptoms of ARI, which may have been influenced by recall bias. Despite these drawbacks, one advantage of our study over earlier research in Ethiopia was the use of DHS data, which includes nationally representative data, enabling generalization of the findings to the entire nation. Furthermore, the majority of prior research in Ethiopia employed symptoms of ARI, fever or diarrhea as end variables; however, in this study, we were able to see the effect of socioeconomic and other demographic determinants on three frequent childhood health problems, as well as mothers’ health care seeking behavior.

## Conclusions and recommendation

This study showed that there is a growing trend of healthcare seeking behavior for diarrhea, fever and ARI symptoms from 2000 to 2016 in Ethiopia. However, there is persistent inequity in healthcare seeking behavior among mothers having under –five children with equity dimension. There is an association of healthcare seeking behavior suffering from diarrhea, fever and symptoms of ARI with various demographic, parental and household characteristics. However, inequities in healthcare seeking behavior persisted in terms of wealth index, educational attainment, residential location, and subnational areas.

To improve health-seeking behavior and reduce inequities in Ethiopia, the government should develop targeted strategies to reach children from diverse socioeconomic backgrounds and administrative areas. Expanding the Community-Based Health Insurance (CBHI) program to provide subsidies or free healthcare for low-income families is crucial. Additionally, implementing community-based education programs focusing on child health, nutrition, disease prevention, and recognizing illness symptoms will enhance public awareness. Strengthening healthcare infrastructure in remote and underserved areas by building more health posts and deploying mobile health clinics should be prioritized. Special attention must also be given to increasing access to antenatal care (ANC) and health facility delivery services. Finally, promoting livelihood programs such as microfinance, vocational training, and social protection schemes will economically empower mothers, contributing to better child health outcomes.

## List of abbreviations:

ANC;: Ante Natal Care
ARI: Acute Respiratory Infection
CDC: Centers for Disease Control and Prevention
CI: Confidence Interval
D: Difference
EAs: Enumerator Areas
EDHS: Ethiopian Demographic and Health survey
HEAT: Health Equity Assessment Toolkit
HEM: Health Equity Monitor
IRB: Institution Review Board
KR: Kid Record
PAF: Population Attributable Fraction
PAR: Population Attributable Risk
R: Ratio
RCI: Relative Concentration Index
SDGs: Sustainable Development Goals
ACI: Absolute Concentration Index
UNICEF: United Nationals Children’s Emergency Fund
WHO: World Health Organization
